# Copy Number Variants Increasing Risk for Schizophrenia: Shared and Distinct Effects on Brain Morphometry and Cognitive Performance

**DOI:** 10.1016/j.bpsgos.2022.10.006

**Published:** 2022-10-29

**Authors:** Xavier Caseras, Sophie E. Legge, Matthew Bracher-Smith, Richard Anney, Michael J. Owen, Valentina Escott-Price, George Kirov

**Affiliations:** Medical Research Council Centre for Neuropsychiatric Genetics and Genomics, Division of Psychological Medicine and Clinical Neurosciences, School of Medicine, Cardiff University, Cardiff, Wales, United Kingdom

**Keywords:** Brain morphometry, Cognition, Copy number variation, Rare variants, Schizophrenia

## Abstract

**Background:**

Copy number variations (CNVs) conferring risk for mental disorders are associated with brain changes and cognitive deficits. However, whether these effects are shared or distinct across CNVs remains untested. Here we compared the effects on brain morphometry and cognitive performance across CNVs with shared psychiatric liability.

**Methods:**

Unaffected and unrelated participants of White British and Irish ancestry were drawn from the UK Biobank. After quality control, we retained 31,941 participants not carrying any damaging CNVs and 202 participants carrying one CNV increasing risk for schizophrenia. Using regression analyses, we tested the association between brain morphometry and cognitive performance with CNV carrying status and compared these effect sizes across CNVs using *z* test for the equality of regression coefficients. Equation modeling was used to examine the mediation of brain phenotypes on the association between CNVs and cognitive performance.

**Results:**

We detected different patterns of association between CNVs and brain morphometry and cognitive abilities. Comparing across CNVs, 1q21.1 deletion showed the strongest association with surface area in frontal lobe (β = −1.03, *p* = 4 × 10^−8^; β = −0.81, *p* = .00001) and performance in digit memory (β = −1.58, *p* = .00003), while 1q21.1 duplication showed the strongest association with volume of the putamen (β = −0.70, *p* = .0004) and reaction time (β = −1.14, *p* = .000002). We also showed that even when 2 CNVs were associated with performance in the same cognitive ability, these associations were mediated by different brain changes.

**Conclusions:**

Despite sharing similar psychiatric liability, the CNVs under study appeared to have different effects on brain morphometry and on performance in cognitive abilities, suggesting the existence of distinctive neurobiological pathways into the same clinical phenotypes.

Several recurrent copy number variations (CNVs), i.e., deletions or duplications of large DNA segments resulting from nonallelic homologous recombination at meiosis, have been identified as risk factors for neurodevelopmental disorders (1), with a small subgroup of these (13 to date) shown to also increase risk for schizophrenia (2,3). Improving our understanding of the shared and distinct phenotypic effects among these CNVs provides a unique opportunity to advance our knowledge of the neurobiological mechanisms behind mental disorders [e.g. (3, 4, 5, 6)].

Previous research has shown these CNVs to have a significant effect on brain morphometry and cognition, with carriers showing an apparent diversity of effects on subcortical and cortical morphometry (7, 8, 9, 10), but a relatively common general decrease in cognitive abilities (9,11, 12, 13, 14). Importantly, previous studies have also shown some of the brain changes reported in carriers to partly mediate the association between carrier status and cognitive performance (7,9,11), informing about potential neurobiological pathways linking genetic risk and clinical phenotypes. However, the extent to which these CNVs show distinctive effects on brain and/or cognition, indicating potential different neurobiological pathways into the same clinical phenotypes, remains unresolved. So far, studies comparing across different CNVs included small sample sizes (7,8), descriptively collated data from independent single studies, or used methodologies not optimized to allow these comparisons (7,8,10,15).

In this study we aimed to investigate the association between carrying damaging CNVs and changes in brain morphometry and cognitive abilities in a sample of unaffected participants, placing the emphasis on cross-CNV comparisons. To this aim, we focused on the subset of 13 CNVs that to date have been shown not only to significantly increase risk for neurodevelopmental disorders but also for schizophrenia (2,3), potentially representing a more homogeneous CNV group with regard to their psychiatric liability. We were also interested in examining whether the potential association between these CNVs and specific cognitive abilities could be mediated by different brain phenotypes (i.e., neurobiological pathways).

## Methods and Materials

### Participants

We used a subsample of UK Biobank participants (https://www.ukbiobank.ac.uk/) from whom brain magnetic resonance imaging (MRI) anatomical images (T1) were available at the time of the analyses (*n* = 47,927). From these, we retained only unrelated participants (estimated kinship coefficient <0.0442; i.e., third-degree relative, coefficient of relatedness <12.5%, *n* = 1489 lost to relatedness) of British/Irish ancestry (based on the first 3 population components compared with 1000 Genomes Project phase 3 superpopulation ancestries, *n* = 3998 lost to ancestry). We then excluded participants who self-reported to have received a diagnosis of schizophrenia or bipolar disorder by a doctor or for whom hospital records for such diagnoses plus schizoaffective or “other psychosis” were documented (*n* = 472). Further exclusion criteria included evidence (self-reported or hospital records) of learning disability or of neurological conditions that could affect brain morphometry (*n* = 346) (Table S1 in Supplement 2). After all of the above genetic and health exclusion criteria, we retained 34,862 participants for whom the brain metrics of interest were available.

All participants provided informed consent to participate in UK Biobank. Ethical approval was granted to the UK Biobank project by the North West Multi-centre Research Ethics Committee. Data were released to us after application project reference 17044.

### Genotyping and CNV Calling

Genotyping was performed using the Affymetrix UK BiLEVE Axiom (Thermo Fisher Scientific) array on the initial 50,000 participants and the Affymetrix UK Biobank Axiom (Thermo Fisher Scientific) array for the remaining participants. The two arrays are extremely similar (with over 95% common content). Sample processing at UK Biobank is described in their documentation (https://biobank.ndph.ox.ac.uk/showcase/refer.cgi?id=155583).

CNV calling was conducted following the same procedure as described in a previous study (12). Briefly, normalized signal intensity, genotype calls, and confidences were generated using approximately 750,000 biallelic markers that were further processed with PennCNV-Affy software (16). Individual samples were excluded if they had >30 CNVs, a waviness factor >0.03 or < −0.03 or call rate <96%. Individual CNVs were excluded if they were covered by <10 probes or had a density coverage of <1 probe per 20,000 base pairs.

The list of CNVs considered in this study, their prevalence in our sample, and the genomic coordinates of their critical regions along with their breakpoints are presented in Table S2 in Supplement 2. These were manually inspected to confirm that they met our CNV calling criteria: We required a CNV to cover more than half of the critical interval and to include the key genes in the region (if known) or, in the case of single-gene CNVs, the deletions to intersect at least one exon and the duplications to cover the whole gene.

Nine carriers of one of our target CNVs also carried at least one other damaging CNV [the criteria for defining damaging CNV has been previously fully described (12)] and were excluded from our analyses. Only 6 of the preselected CNVs were present in more than 5 participants and were taken forward into our analyses (Table 1). As a control comparison group, we used individuals who carried none of the 90 CNVs defined as damaging due to their association with neurodevelopmental disorders (17,18) (non-CNV carriers, *n* = 31,941).

**Table 1:** Sociodemographic Characteristics in CNV Carriers and Non-CNV Carriers

CNV	*n*	Age, Years, Mean (SD)	Sex, Female, %	Qualification^b^, Mean (SD)	Income^c^, Mean (SD)	Townsend^d^, Mean (SD)
1q21.1del	10	58.5 (6.4)	50.0%	2.59 (1.54)	1.82 (1.51)	−0.28 (3.94)
1q21.1dup	17	62.3 (6.4)	70.6%	1.89 (0.92)	2.90 (0.99)	−1.31 (2.79)
*NRXN1*del	11	63.4 (6.2)	54.5%	2.44 (1.33)	2.64 (1.28)	−2.80 (2.16)
15q11.2del	106	63.9 (7.2)	50.9%	2.98 (1.80)	2.17 (1.55)	−1.93 (2.52)
16p13.11dup	45	64.6 (6.2)	46.7%	2.98 (1.94)	1.82 (2.28)	−2.02 (2.50)
16p12.1del	13	59.7 (6.6)	69.2%	2.62 (1.89)	1.54 (2.84)	−1.71 (3.15)
Noncarriers^a^	31,941	63.7 (7.5)	52.7%	2.24 (1.54)	2.54 (1.88)	−1.98 (2.66)

CNV, copy number variation; del, deletion; dup, duplication.

a Noncarriers were participants not carrying any of the 90 CNVs identified as damaging by previous research.

b Qualification was highest educational qualification achieved (lower number indicates higher qualification with 1 equivalent to university or college degree).

c Income was defined as household annual income categorized by the UK Biobank in 5 bands: <£18,000, £18,000–£30,999, £31,000–£51,999, £52,000–£100,000, and >£100,000.

d Townsend Deprivation index was assigned to participants based on their postal code at the time of recruitment (higher values indicate higher deprivation, with negative values related to affluent areas).

### Brain Imaging and Cognitive Data

Brain images were acquired using Siemens MAGNETOM Skyra (Siemens Corp.) 3T MRI scanners in UK Biobank imaging centers using identical acquisition protocols. T1-weighted brain images were processed using different procedures, and a basic quality control was run on the raw images (documentation on data acquisition and processing is freely available from UK Biobank at https://biobank.ctsu.ox.ac.uk/crystal/ukb/docs/brain_mri.pdf). For our project, we focused on estimates of subcortical volumes (in mm^3^), mean cortical thickness (in mm), and surface area (in mm^2^) for each gyrus based on the Desikan-Killiany atlas parcellation obtained via FreeSurfer v.5.3 software (https://surfer.nmr.mgh.harvard.edu/). Average cortical thickness and total surface area were calculated for the frontal, parietal, temporal, and occipital lobes and the cingulate cortex. To avoid error values due to deficient segmentation of tissue types or parcellation into gyri, extreme values, defined as ±3 SDs from the group mean, were removed from the analyses.

On the date of their scan, UK Biobank participants were invited to complete a cognitive test battery assessing different cognitive skills involving reasoning, memory, and speed processing (https://biobank.ndph.ox.ac.uk/showcase/label.cgi?id=100026) that have shown moderate-to-high indices of validity (19). Based on their distribution being closer to normality in our sample, from these we selected the following: associative learning (number of words paired correctly), card pairs matching (errors on second round), digit memory (maximum number of digits remembered correctly), fluid intelligence (total score), reaction time (mean time to correctly identify matches), trail making numeric (duration to complete), trail making alphanumeric (duration to complete), symbol digit matches (correct responses), matrix patterns (number correct), and tower rearrangement (number correct).

### Analyses

Regression analyses were used to test the association between CNV carrier status (predictor) and brain and cognitive phenotypes (outcome), while controlling for the effect of several confounders (i.e., age, sex, age × sex, testing center, date attended, and the first 10 population stratification principal components; regression models for brain phenotypes also included intracranial volume; *x*, *y*, and *z* brain position in the scanner; scanner table position; and head motion estimated from the resting-state functional MRI scan). The outcome variables were *z*-transformed before the analyses, and carrier status was coded as 0 noncarrier versus 1 carrier. Therefore, the regression coefficients (β coefficients) represent the change of the outcome variable in standard deviations associated with carrying CNV under analysis. Bonferroni correction for the number of independent tests was applied for subcortical volumes (42 tests, *p* < .001), cortical measures (72 tests, *p* < .0007), and cognitive skills (60 tests, *p* < .0008). To compare the effects on brain and cognition across CNVs, the β coefficients from the above regression analyses were compared using *z* test for the equality of regression coefficients.

To examine the ability of brain morphometry to account for the association between CNV carrier status and cognitive performance, mediation analyses were conducted in a structural equation modeling framework using the Lavaan package in R (20). We applied this analysis to cognitive tests that showed significant association (*p* < .05) with at least 2 CNVs. For each of these, we entered as mediators those brain measures more strongly associated with performance on that cognitive test. To identify those, we first ran regression models for each cognitive test as outcome and all brain phenotypes and covariates as predictors; we retained for the mediation analyses those brain phenotypes that were significant in these models (Table S3 in Supplement 2).

## Results

### Brain Morphometry

#### Subcortical Volumes

**Figure 1:**
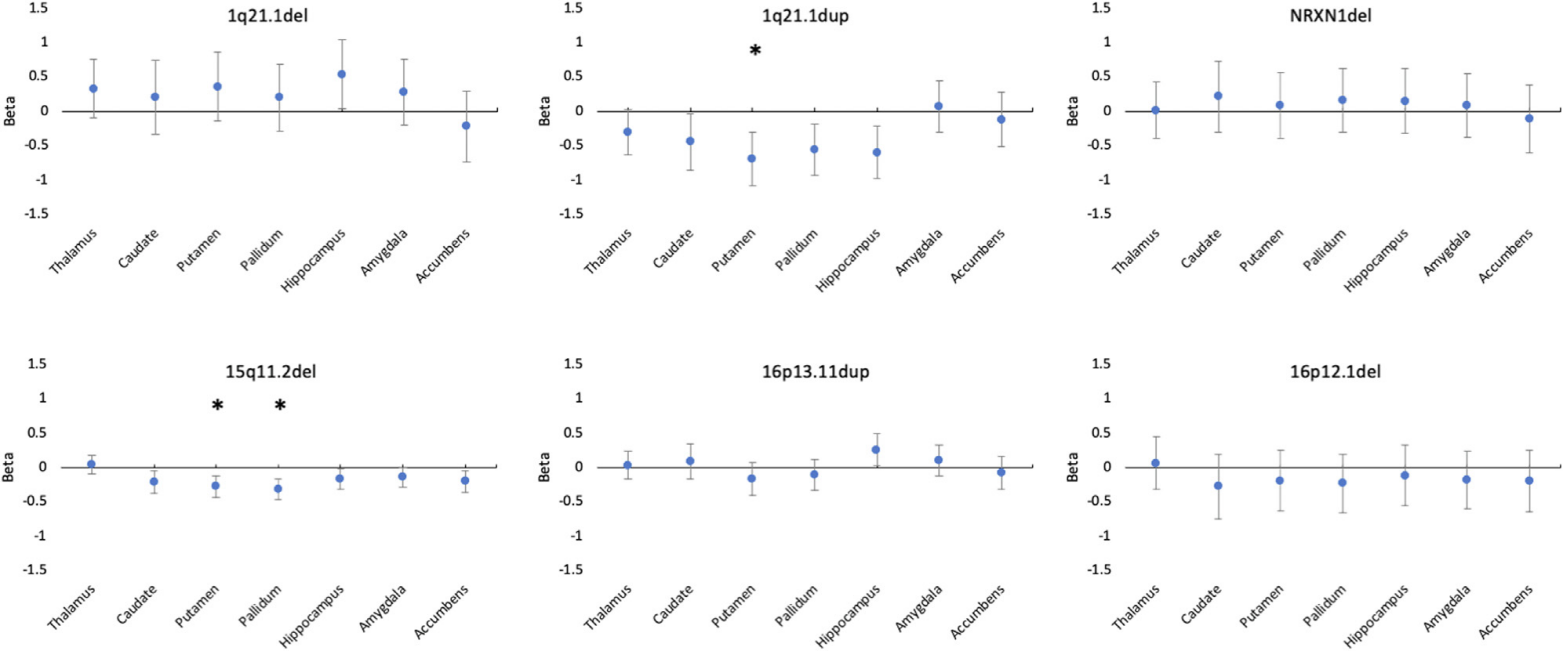
Association (β coefficients from regression analyses) between carrier status for each schizophrenia copy number variation present in the sample and subcortical volumes. Positive values indicate increased volume in carriers; negative values indicate decreased volume in carriers. Error bars represent the 95% confidence interval of the β coefficients. ∗Significant association after correction for multiple testing (*p* < .001). del, deletion; dup, duplication.

**Figure 2:**
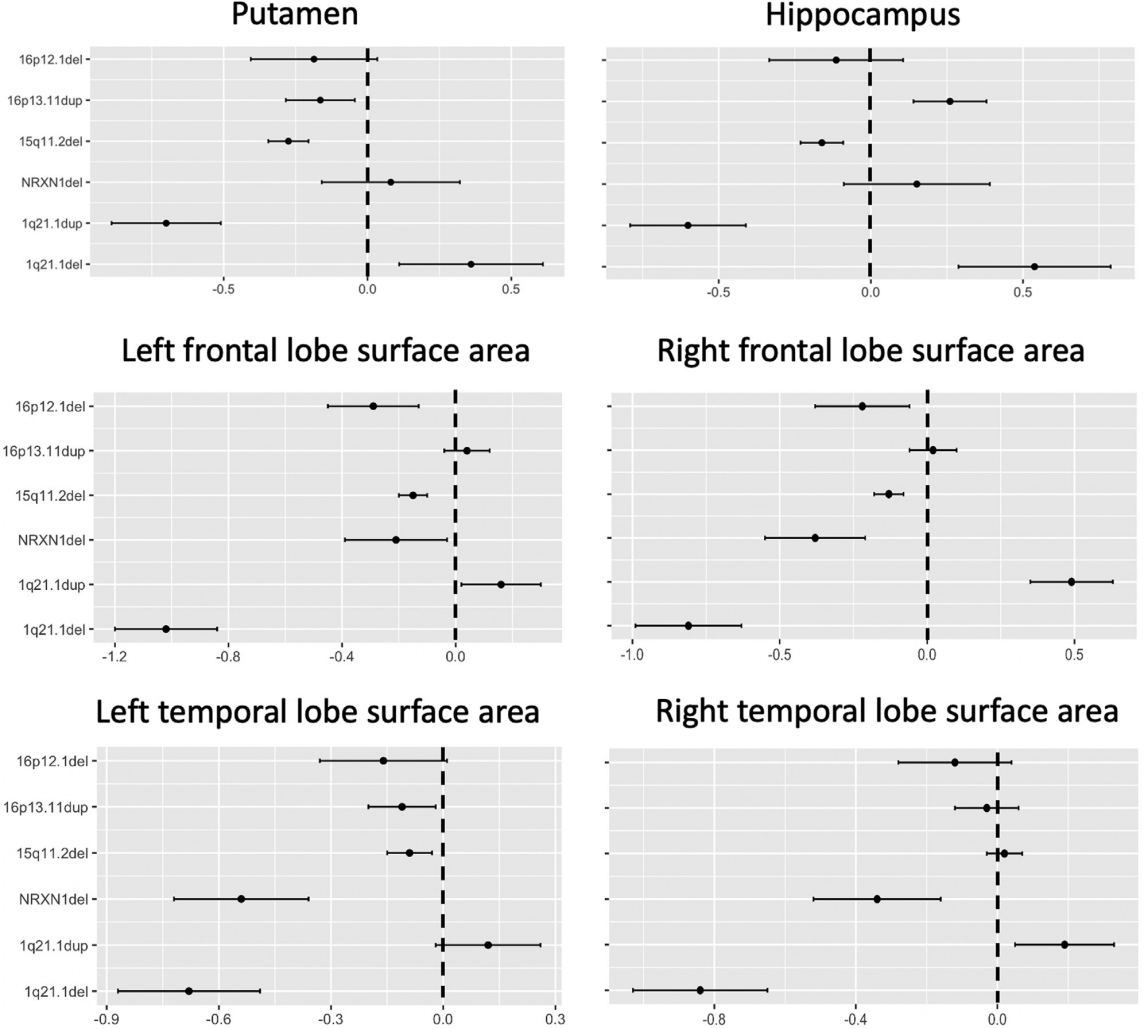
Effect sizes (β coefficients) for the effect of carrying a single schizophrenia copy number variation (CNV) on brain morphometric markers that showed significant association with carrying status (carrier vs. non-CNV carrier). This figure allows direct comparison across different schizophrenia CNVs. β coefficients indicate the difference in standard error between carrying a specific CNV and noncarrying any pathological CNV. Error bars represent the standard error of β coefficients. del, deletion; dup, duplication.

The 15q11.2 deletion and 1q21.1 duplication showed several significant associations with subcortical volumes at *p* < .05, in all cases indicating reduced volume in carriers. The 1q21.1 deletion and 16p13.11 duplication carriers showed increased hippocampal volume at *p* < .05. Only the associations of 15q11.2 deletion and 1q21.1 duplication with the volume of the putamen and of 15q11.2 deletion with the volume of the pallidum remained significant after correction for multiple testing (*p* < .001) (Figure 1; Table 2). The comparison of β coefficients across these associations showed the 1q21.1 duplication to have the largest negative effect on putamen (vs. 15q11.2 deletion [*p* = .04], vs. 16p13.11 duplication [*p* = .01], and vs. 16p12.1 deletion [*p* = .07]) and hippocampus (vs. 15q11.2 deletion [*p* = .03] and vs. 16p12.1 deletion [*p* = .09]) (Figure 2). As per 15q11.2 deletion, none of the β coefficients found were significantly larger than those of any other CNV.

**Table 2:** Association Between CNV Carrier Status and Subcortical Volumes

Volume	1q21.1del, *n* = 10	1q21.1dup, *n* = 17	*NRXN1*del, *n* = 11	15q11.2del, *n* = 106	16p13.11dup, *n* = 45	16p12.1del, *n* = 13
Thalamus	0.33 (*p* > .1)	−0.30 (*p* = .07)	0.01 (*p* > .1)	0.047 (*p* > .1)	0.03 (*p* > .1)	0.06 (*p* > .1)
Caudate	0.20 (*p* > .1)	−0.44 (*p* = .03)	0.21 (*p* > .1)	−0.20 (*p* = .01)	0.09 (*p* > .1)	−0.27 (*p* > .1)
Putamen	0.36 (*p* > .1)	−0.70 (*p* < .001)^a^	0.08 (*p* > .1)	−0.27 (*p* < .001)^a^	−0.16 (*p* > .1)	−0.18 (*p* > .1)
Pallidum	0.19 (*p* > .1)	−0.56 (*p* = .003)	0.16 (*p* > .1)	−0.31 (*p* < .001)^a^	−0.10 (*p* > .1)	−0.22 (*p* > .1)
Hippocampus	0.53 (*p* = .03)	−0.60 (*p* = .002)	0.15 (*p* > .1)	−0.16 (*p* = .04)	0.26 (*p* = .03)	−0.11 (*p* > .1)
Amygdala	0.27 (*p* > .1)	0.06 (*p* > .1)	0.08 (*p* > .1)	−0.14 (*p* = .06)	0.10 (*p* > .1)	−0.17 (*p* > .1)
Accumbens	−0.21 (*p* > .1)	−0.12 (*p* > .1)	−0.11 (*p* > .1)	−0.19 (*p* = .01)	−0.07 (*p* > .1)	−0.19 (*p* > .1)

Association is reported as β coefficient (*p* value).

CNV, copy number variation; del, deletion; dup, duplication.

a Significant after Bonferroni correction based on 7 brain factors explaining >95% of the variance × 6 schizophrenia CNVs = 42 tests.

Carriers of the 1q21.1 deletion and duplication showed the opposite direction of effect (positive for deletion, negative for duplication) in all subcortical volumes except amygdala and accumbens. These differences reached significance in hippocampus (β = −1.04, *p* = .0003), putamen (β = −1.02, *p* = .0005), thalamus (β = −0.60, *p* = .02), and pallidum (β = −0.65, *p* = .03) (Table S4 in Supplement 2).

#### Cortical Thickness and Surface Area

**Figure 3:**
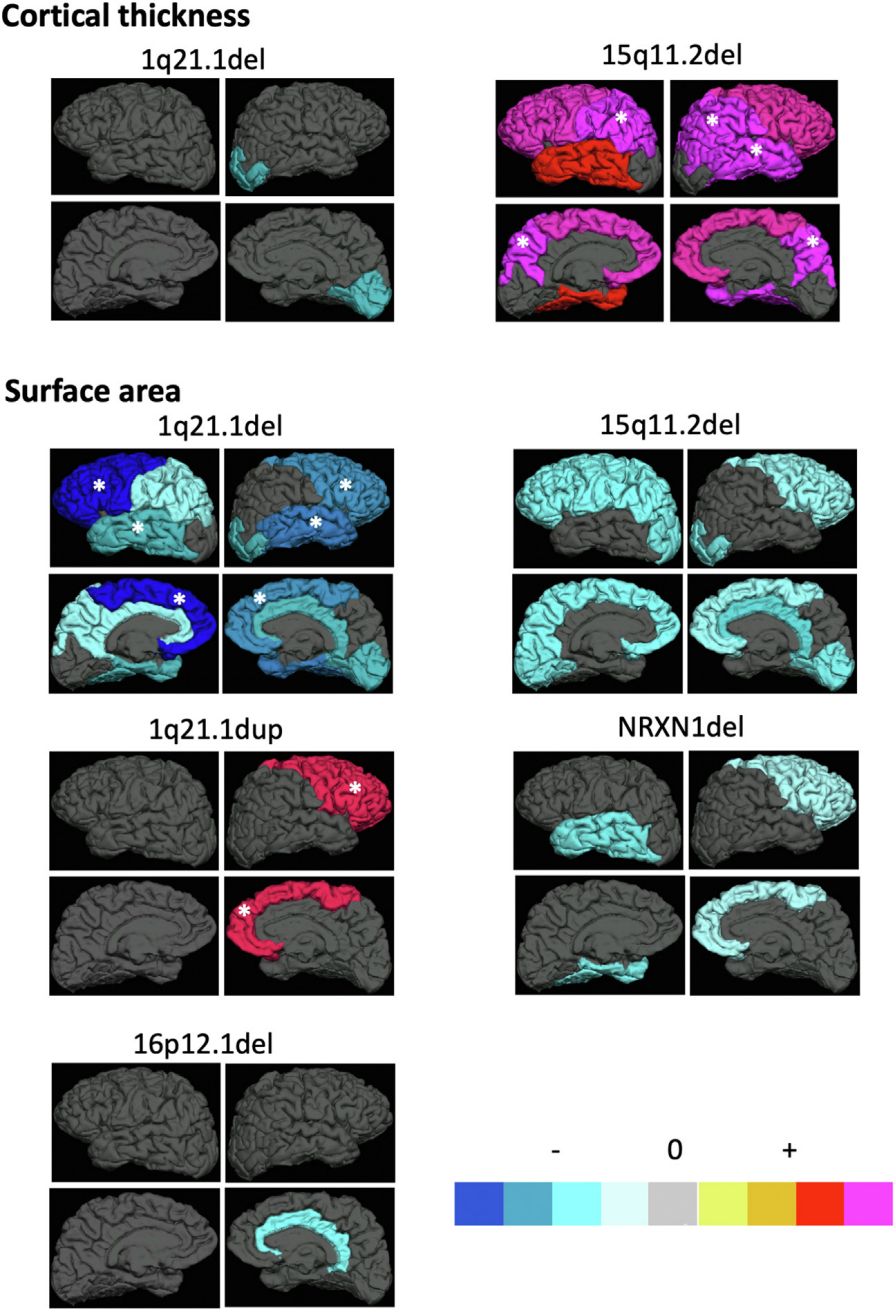
Association (β coefficients from regression analyses) between carrier status for each schizophrenia copy number variation present in the sample and average cortical thickness (top panels) and total surface area (bottom panels) in left and right frontal, parietal, temporal, and occipital lobes and cingulate cortex. Warmer color indicates increased thickness/area in carriers; cooler color indicates reduced thickness/area in carriers. Only brain areas with at least nominal significant results are colored. ∗Associations where the significant result survives correction for multiple testing (*p* < .0007). del, deletion; dup, duplication.

Carrying the 15q11.2 deletion was associated with widespread thicker cortex in all lobes but the occipital; however, only the association with both parietal lobes and right temporal lobe remained significant after correction for multiple testing (*p* < .0007) (Figure 3; Table 3). The comparison of β coefficients across CNVs, though, showed the effects of 15q11.2 deletion to not be significantly different from those of most other CNVs.

**Table 3:** Association Between CNV Carrier Status and Cortical Thickness and Surface Area

	1q21.1del, *n* = 10		1q21.1dup, *n* = 17		*NRXN1*del, *n* = 11		15q11.2del, *n* = 106		16p13.11dup, *n* = 45		16p12.1del, *n* = 13	
	Left	Right	Left	Right	Left	Right	Left	Right	Left	Right	Left	Right
Thickness												
Frontal	0.25 (*p* > .1)	−0.06 (*p* > .1)	−0.01 (*p* > .1)	0.25 (*p* > .1)	0.16 (*p* > .1)	0.35 (*p* > .1)	0.26 (*p* = .006)	0.24 (*p* = .012)	−0.04 (*p* > .1)	−0.13 (*p* > .1)	0.04 (*p* > .1)	−0.29 (*p* > .1)
Parietal	0.22 (*p* > .1)	−0.05 (*p* > .1)	−0.08 (*p* > .1)	−0.15 (*p* > .1)	0.39 (*p* > .1)	0.31 (*p* > .1)	0.39 (*p* < .0007)^a^	0.40 (*p* < .0007)^a^	−0.03 (*p* > .1)	−0.04 (*p* > .1)	0.07 (*p* > .1)	−0.09 (*p* > .1)
Temporal	−0.19 (*p* > .1)	−0.06 (*p* > .1)	−0.29 (*p* > .1)	0.15 (*p* > .1)	0.52 (*p* = .08)	0.35 (*p* > .1)	0.17 (*p* = .07)	0.38 (*p* < .0007)^a^	0.14 (*p* > .1)	−0.01 (*p* > .1)	−0.24 (*p* > .1)	−0.25 (*p* > .1)
Occipital	−0.34 (*p* > .1)	−0.67 (*p* = .03)	−0.17 (*p* > .1)	0.29 (*p* > .1)	0.34 (*p* > .1)	−0.11 (*p* > .1)	0.08 (*p* > .1)	0.11 (*p* > .1)	−0.04 (*p* > .1)	−0.002 (*p* > .1)	−0.17 (*p* > .1)	0.03 (*p* > .1)
Cingulate	−0.36 (*p* > .1)	0.13 (*p* > .1)	0.27 (*p* > .1)	0.13 (*p* > .1)	0.24 (*p* > .1)	0.24 (*p* > .1)	0.01 (*p* > .1)	−0.01 (*p* > .1)	0.04 (*p* > .1)	−0.21 (*p* > .1)	0.01 (*p* > .1)	−0.08 (*p* > .1)

Area												
Frontal	−1.03 (*p* < .0007)^a^	−0.81 (*p* < .0007)^a^	0.16 (*p* > .1)	0.49 (*p* < .0007)^a^	−0.21 (*p* > .1)	−0.38 (*p* = .02)	−0.15 (*p* = .007)	−0.13 (*p* = .02)	0.04 (*p* > .1)	0.02 (*p* > .1)	−0.29 (*p* = .07)	−0.22 (*p* > .1)
Parietal	−0.47 (*p* = .02)	−0.31 (*p* > .1)	−0.02 (*p* > .1)	0.01 (*p* > .1)	−0.18 (*p* > .1)	−0.24 (*p* > .1)	−0.15 (*p* = .01)	−0.08 (*p* > .1)	−0.08 (*p* > .1)	−0.005 (*p* > .1)	−0.03 (*p* > .1)	−0.02 (*p* > .1)
Temporal	−0.69 (*p* < .0007)^a^	−0.84 (*p* < .0007)^a^	0.12 (*p* > .1)	0.19 (*p* > .1)	−0.54 (*p* = .004)	−0.34 (*p* = .06)	−0.09 (*p* > .1)	0.02 (*p* > .1)	−0.11 (*p* > .1)	−0.03 (*p* > .1)	−0.16 (*p* > .1)	−0.12 (*p* > .1)
Occipital	−0.37 (*p* > .1)	−0.41 (*p* > .1)	0.02 (*p* > .1)	0.19 (*p* > .1)	−0.13 (*p* > .1)	0.16 (*p* > .1)	−0.17 (*p* = .03)	−0.17 (*p* = .02)	−0.21 (*p* = .09)	−0.11 (*p* > 0.1)	−0.01 (*p* > 0.1)	−0.08 (*p* > 0.1)
Cingulate	−0.47 (*p* = .04)	−0.66 (*p* = .007)	0.02 (*p* > .1)	0.08 (*p* > .1)	−0.15 (*p* > .1)	−0.06 (*p* > .1)	−0.10 (*p* > .1)	−0.18 (*p* = .01)	0.11 (*p* > .1)	−0.16 (*p* > .1)	−0.28 (*p* > .1)	−0.43 (*p* = .04)

Association is reported as β coefficient (*p* value).

CNV, copy number variation; del, deletion; dup, duplication.

a Significant after Bonferroni correction based on 12 brain factors explaining >95% of the variance × 6 schizophrenia CNVs = 72 tests.

Several schizophrenia copy number variations showed nominal associations with surface area indicating reduced area in carriers except for 1q21.1 duplication, which showed the opposite direction of effect. However, only the effects of 1q21.1 deletion on frontal and temporal lobes bilaterally and of 1q21.1 duplication on the right frontal lobe survived correction for multiple testing (*p* < .0007) (Figure 3; Table 3). The comparison of β coefficients showed that the negative effects of 1q21.1 deletion on left and right frontal lobes were significantly stronger than those of any other CNVs (vs. 15q11.2 deletion [*p* = 1 × 10^−5^ and *p* = .0005, left and right, respectively], vs. 16p12.1 deletion [*p* = .002 and *p* = .014, left and right, respectively], and vs. *NRXN1* deletion on the left frontal lobe [*p* = .001], but not on the right [*p* = .1]) (Figure 2). Likewise, the negative effects of 1q21.1 deletion on left and right temporal lobe were among the largest detected (vs. 16p12.1 deletion [*p* = .04 and *p* = .003, left and right, respectively] and vs. *NRXN1* deletion on the right [*p* = .06], but not the left [*p* > .1]) (Figure 2).

Results from a more fine-grain parcellation of the cortex in gyri based on the Desikan-Killiany atlas (Table S5 in Supplement 2) confirmed the above results: widespread positive association of 15q11.2 deletion with thickness predominantly in frontal and parietal cortices, albeit with effect sizes not larger than those of other CNVs. Also, 1q21.1 deletion showed its stronger effects in gyri within the frontal and temporal lobes, these being among the strongest effects found across the board. Finally, 1q21.1 deletion and duplication carriers showed again mostly opposing directions of effects on frontal and temporal surface areas (reductions in deletion carriers and increases in duplication carriers) (Table S6 in Supplement 2).

### Cognitive Performance

**Figure 4:**
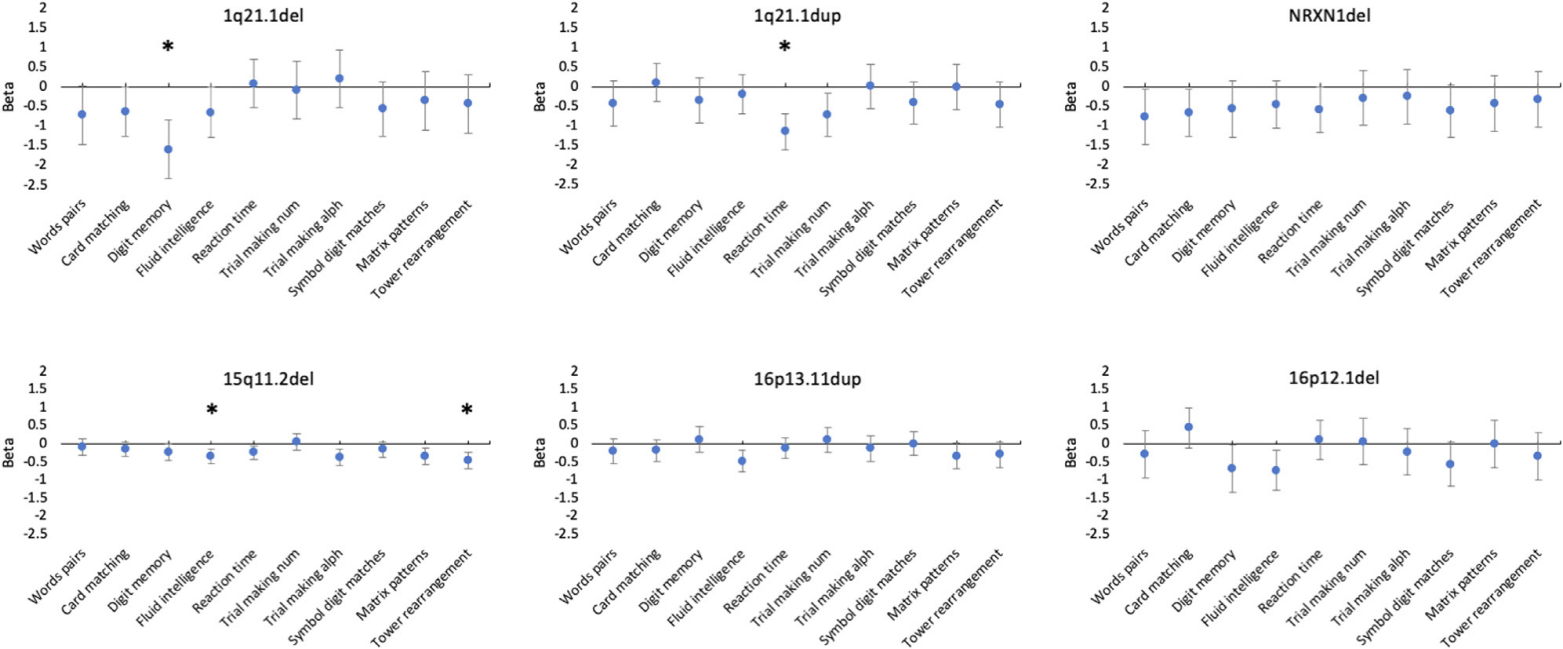
Association (β coefficients from regression analyses) between carrier status for each schizophrenia copy number variation present in the sample and performance in cognitive tests. In all cases, positive values indicate higher performance in carriers, and negative values indicate lower performance in carriers. Error bars represent the 95% confidence interval of the β coefficients. ∗Significant association after correction for multiple testing (*p* < .008). alph, alphanumeric; del, deletion; dup, duplication; num, numeric.

**Figure 5:**
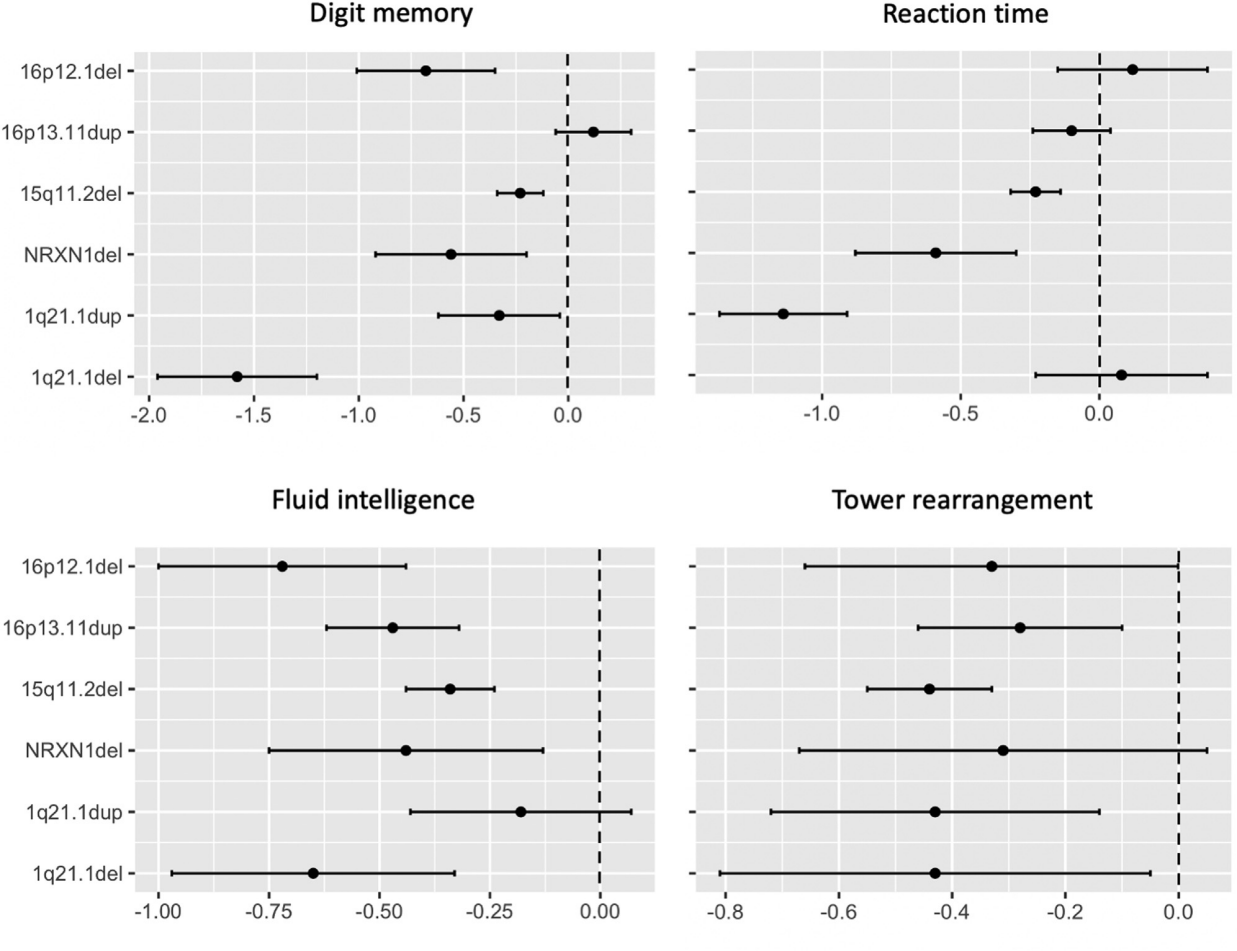
Effect sizes (β coefficients) for the effect of carrying a single schizophrenia copy number variation (CNV) on performance in cognitive tests that showed significant association with carrying status (carrier vs. non-CNV carrier). This figure allows direct comparison across different schizophrenia CNVs. β coefficients indicate the difference in standard error between carrying a specific CNV and noncarrying any pathological CNV. Error bars represent the standard error of the β coefficients. del, deletion; dup, duplication.

Multiple significant associations at *p* < .05 were found between CNVs and cognitive abilities (Figure 4; Table 4), in all cases indicating poorer performance in carriers. However, only 4 of these associations survived correction for multiple testing: 1q21.1 deletion with digit memory (β = −1.58, *p* = .00003), 1q21.1 duplication with reaction time (β = −1.14, *p* = .000002), and 15q11.2 deletion with fluid intelligence and tower rearrangement (β = −0.34, *p* = .0005 and β = −0.44, *p* = .0001, respectively). The comparison of β coefficients (Figure 5) showed the 1q21.1 deletion to have the largest effect on digit memory (vs. 1q21.1 duplication [*p* = .008], vs. *NRXN1* deletion [*p* = .05], vs. 15q11.2 deletion [*p* = .001], and vs. 16p12.1 deletion [*p* = .07]); the 1q21.1 duplication was shown to have one of the largest effects on reaction time (vs. 15q11.2 deletion [*p* = .0004] and vs. 16p13.11 duplication [*p* = .0001], except compared with *NRXN1* deletion [*p* > .1]). The effects of 15q11.2 deletion on fluid intelligence and tower rearrangement were not statistically larger than those shown by any other CNVs (all *p* > .2).

**Table 4:** Association Between CNV Carrier Status and Cognitive Skills

Cognitive Skills Test	1q21.1del, *n* = 10	1q21.1dup, *n* = 17	*NRXN1*del, *n* = 11	15q11.2del, *n* = 106	16p13.11dup, *n* = 45	16p12.1del, *n* = 13
Word Pairs	−0.71 (*p* = .06)	−0.42 (*p* > .1)	−0.75 (*p* = .03)	−0.07 (*p* > .1)	−0.19 (*p* > .1)	−0.28 (*p* > .1)
Card Pairs Matching	−0.62 (*p* =.05)	0.10 (*p* > .1)	−0.65 (*p* = .03)	−0.14 (*p* > .1)	−0.16 (*p* > .1)	0.44 (*p* > .1)
Digit Memory	−1.5 (*p* < .0008)^a^	−0.33 (*p* > .1)	−0.56 (*p* > .1)	−0.23 (*p* = .05)	0.12 (*p* > .1)	−0.68 (*p* = .04)
Fluid Intelligence	−0.65 (*p* = .04)	−0.18 (*p* > .1)	−0.44 (*p* > .1)	−0.34 (*p* < .0008)^a^	−0.47 (*p* = .002)	−0.72 (*p* = .01)
Reaction Time	0.08 (*p* > .1)	−1.1 (*p* < .0008)^a^	−0.59 (*p* = .04)	−0.23 (*p* = .01)	−0.10 (*p* > .1)	0.12 (*p* > .1)
Trail Making Num	−0.08 (*p* > .1)	−0.71 (*p* = .01)	−0.28 (*p* > .1)	0.06 (*p* > .1)	0.12 (*p* > .1)	0.07 (*p* > .1)
Trail Making Alph	0.21 (*p* > .1)	0.009 (*p* > .1)	−0.25 (*p* > .1)	−0.36 (*p* = .001)	−0.12 (*p* > .1)	−0.21(*p* > .1)
Symbol Digit Matches	−0.56 (*p* > .1)	−0.41 (*p* > .1)	−0.62 (*p* = .06)	−0.14 (*p* > .1)	0.01 (*p* > .1)	−0.55 (*p* = .07)
Matrix Patterns	−0.35 (*p* > .1)	−0.001 (*p* > .1)	−0.42 (*p* > .1)	−0.33 (*p* = .005)	−0.33 (*p* = .06)	−0.01 (*p* > .1)
Tower Rearrangement	−0.43 (*p* > .1)	−0.43 (*p* > .1)	−0.32 (*p* > .1)	−0.44 (*p* < .0008)^a^	−0.28 (*p* > .1)	−0.33 (*p* > .1)

Association is reported as β coefficient (*p* value).

Alph, alphanumeric; CNV, copy number variation; del, deletion; dup, duplication; num, numeric.

a Significant after Bonferroni correction based on 10 cognitive tests explaining >95% of the variance × 6 schizophrenia CNVs = 60 tests.

### Mediation Analyses

For digit memory, 3 mediation models were independently tested for 1q21.1 deletion, 15q11.2 deletion, and 16p12.1 deletion (Figure S1 in Supplement 1). None of these resulted in any significant mediation effects.

Mediation models for fluid intelligence were tested for 15q11.2 deletion, 16p13.11 duplication, and 16p12.1deletion (Figure S2 in Supplement 1). Hippocampal volume appeared to mediate the association between 15q11.2 deletion (β = −0.009, *p* = .05) and performance in this cognitive test, also showing a trend to mediate this association for 16p13.11 duplication (β = 0.012, *p* = .06). Interestingly, these effects were in the opposite direction.

Mediation models for reaction time were tested for 1q21.1 duplication, *NRXN1* deletion, and 15q11.2 deletion (Figure S3 in Supplement 1). For 1q21.1 duplication, volume of the thalamus (β = 0.019, *p* = .003), pallidum (β = 0.019, *p* = .020), and hippocampus (β = 0.018, *p* = .011) showed significant mediation effects. For 15q11.2 deletion, volume of the pallidum (β = 0.010, *p* = .018) and area of the right cingulum (β = −0.006, *p* = .039) mediated the association with reaction time. No significant effects were found for the model including *NRXN1* deletion.

## Discussion

The main aim of this study was to compare the effects of carrying different CNVs on brain morphometry and cognitive performance and to examine the potential existence of different brain morphometric pathways mediating the association between CNVs and cognitive performance. Our results show that despite selecting a homogeneous group of CNVs with regard to their psychiatric liability, these demonstrate distinctive patterns of associations with brain morphometry and cognitive abilities. Moreover, we also show that even when two CNVs are associated with the same behavioral phenotype (i.e., performance in a cognitive test), these associations are mediated by different brain changes (i.e., neurobiological pathways).

The methods in this study were optimized in several ways to allow for comparison of the effects on brain morphometry and cognitive performance across CNVs. First, as individual CNVs have different penetrance, which would have resulted in different ratios of affected to unaffected participants, by including only healthy participants, we avoided confounding the comparison by reverse causation. Second, by focusing on a large sample drawn from a single cohort population (i.e., participants of British/Irish ancestry living in the United Kingdom), we minimized sociodemographic variability (Table 1). Third, variability in our phenotypes was minimized by applying the same acquisition/processing MRI protocol on data obtained in 3 identical scanners (Siemens MAGNETOM Skyra 3T) and a unique cognitive testing protocol; furthermore, testing site, date of data acquisition, and other potential MRI sources of noise were accounted for in our analyses (21). Finally, we kept the non-CNV carrier group unchanged across the analyses for individual CNVs, allowing a direct comparison of their effect sizes. Whereas some recent studies have compared the effects on brain morphometry and cognition across CNVs (10,15), to our knowledge, this is the first study applying an optimized design to support such a comparison.

Our results show important differences in the association of individual CNVs with brain morphometry. Whereas most rare variants showed mainly small nonsignificant effects on subcortical volumes, 1q21.1 duplication showed its largest associations with volume of the striatum and hippocampus, these effects being statistically larger than those of most other CNVs. Moreover, 1q21.1 duplication and 15q11.2 deletion showed the opposite direction of effect over hippocampal volume (reduction) compared with 1q21.1 deletion and 16p13.11 duplication (increase). This diversity was also evident in the cortex, where 15q11.2 deletion was associated more strongly with cortical thickness, whereas 1q21.1 deletion was associated with surface area; most other CNVs showed weak, sparse associations with either measure. Reductions in surface area associated with 1q21.1 deletion in frontal and temporal lobes were significantly larger than those of most other CNVs. Despite 15q11.2 deletion showing several significant associations with subcortical and cortical measures, the effect sizes were not significantly larger than those of most other CNVs. The fact that 15q11.2 deletion was the most prevalent CNV in our sample and therefore carried the largest statistical power explained the larger number of significant results despite the lack of differences in effect size. This concurs with recent literature showing that 15q11.2 deletion is one of the most prevalent rare variants in the general population, but with a less negative effect on health (22,23).

We also investigated potential dose effects of the deletion and the duplication at 1q21.1, finding several instances where deletion and duplication carriers showed the opposite direction of effect. However, only for hippocampal volume and surface area in right frontal lobe, dose effects were confirmed by both carrier groups being statistically different from non-CNV carriers, replicating previous results from a multicenter study with clinical and nonclinical samples (9).

We also found different association profiles across CNVs with cognitive performance: 15q11.2 deletion was mostly associated with tasks involving executive function and reasoning (i.e., fluid intelligence, tower rearrangement, matrix patterns, and trail making alphanumerical), 1q21.1 duplication was mostly associated with performance on tasks tapping into speed processing (i.e., reaction time and trail making numerical), and 1q21.1 deletion was mostly associated with working memory (i.e., digit memory, card matching, and word pairs). It is also interesting to note that at nominal level (*p* < .05), all CNVs showed some association with cognitive performance, which supports the idea that deeper, more detailed phenotyping of samples should allow a better understanding of the effects of rare variants on observable phenotypes. In this respect, the use of single clinical diagnoses might be limiting progress in the field. The deletion and the duplication CNVs at 1q21.1 showed the strongest detrimental effects on cognition: the deletion on working memory and the duplication on speed processing. The 15q11.2 deletion benefited again from a higher statistical power (i.e., larger number of carriers), but none of the effect sizes found for this CNV were statistically different from those of most other CNVs. Interestingly, in contrast to brain morphometry, no dose effects were observed between the 1q21.1 deletion and duplication for any cognitive ability.

Finally, we showed that different brain morphometric measures (i.e., potentially distinct neurobiological mechanisms) could mediate the association between individual CNVs and cognitive abilities. For example, we found that hippocampal volume partly contributed to the lower performance in reaction time of 15q11.2 deletion carriers, whereas it showed a protective role in carriers of the 16p13.11 duplication. This result highlights the fact that some of the brain changes seen in unaffected carriers of these CNVs—most likely those discordant with changes shown in affected participants—could be protective against developing mental disorders and warrant further investigation.

Some limitations of this study should be highlighted. First, due to our strict selection criteria, the number of carriers for some individual CNVs was limited, and consequently the statistical power to detect significant effects was limited. As such, future research with larger samples of CNV carriers should be able to identify more subtle differences across CNVs. Second, also due to our selection criteria, rarer and potentially more penetrant CNVs were not present in our sample (e.g., 22q11.2 deletion). In this case, a joint effort with a consortium such as ENIGMA (Enhancing Neuro Imaging Genetics through Meta Analysis) is important in accessing these participants. Third, despite that UK Biobank participants are in general healthier, wealthier, and more educated than the general population of the United Kingdom (24) and that our carriers did not differ from noncarriers in these measures, there is the possibility that subthreshold symptoms not accounted for could have partly explained some of the group differences found here. Fourth, we limited our brain measures to metrics of macrostructure and found some CNVs to show very little, if any, association with these. This should not be taken as a suggestion that these rare variants are unrelated to brain biomarkers, but rather that research focusing on other brain phenotypes, such are neurite density, myelin content, or network connectivity, should shed further light on the effects of these CNVs.

In conclusion, we showed that CNVs with a rather homogeneous psychiatric liability have important differential effects on brain phenotypes, with 1q21.1 duplication and 1q21.1 deletion showing the strongest effects, respectively, on the volume of striatum and hippocampus and surface area in frontotemporal cortices. All CNVs investigated showed negative effects on cognitive performance, although the strength of the effects differed across CNVs and cognitive abilities. Even when 2 CNVs were associated with the same cognitive phenotype, this association appeared to be mediated by different brain changes, suggesting the existence of different neurobiological pathways to the same phenotype.

## Supplementary Material

Supplemental Tables 1-6

Key Resources Table
