# Supplementary material for: Copy Number Variants Increasing Risk for Schizophrenia: Shared and Distinct Effects on Brain Morphometry and Cognitive Performance
Source: Biol Psychiatry Glob Open Sci. 2022 Oct 29;3(4):902–11. doi: 10.1016/j.bpsgos.2022.10.006 (PMC10593876; doi:10.1016/j.bpsgos.2022.10.006)
Supplement: Supplementary Information [file mmc1.pdf]

# SUPPLEMENTARY INFORMATION

# Copy Number Variants Increasing Risk for Schizophrenia: Shared and Distinct Effects on Brain Morphometry and Cognitive Performance

Caseras *et al.*

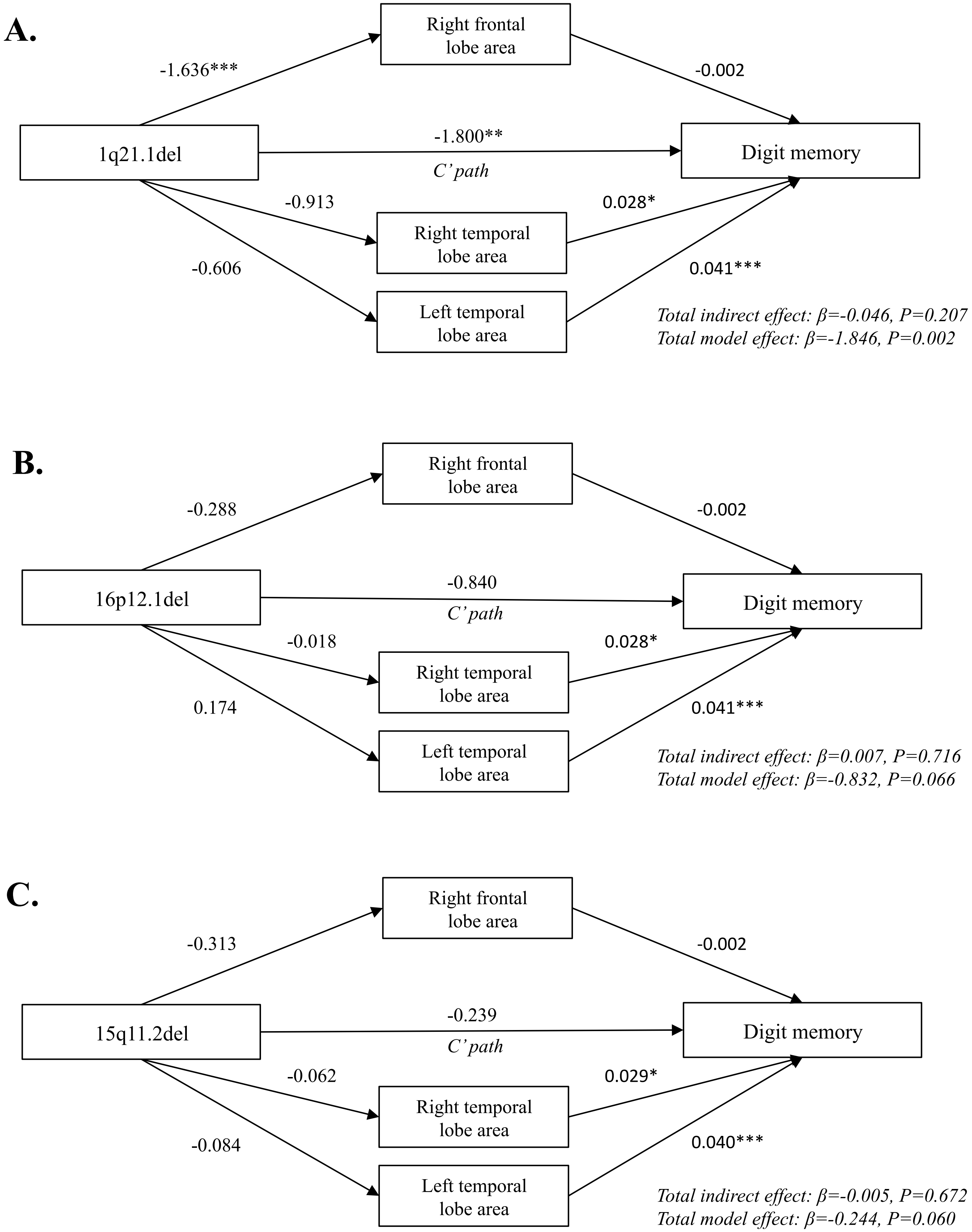


**Supplemental Figure 1**. Mediation analyses for the digit memory task and 1q21.1del (Figure A), 16p12.1del (Figure B), and 15q11.2del (Figure C). Associations are presented as Beta values and significance denoted by * ($p<0.05$), **($p<0.01$), ***($p<0.001$). The C' path refers to the direct effect.

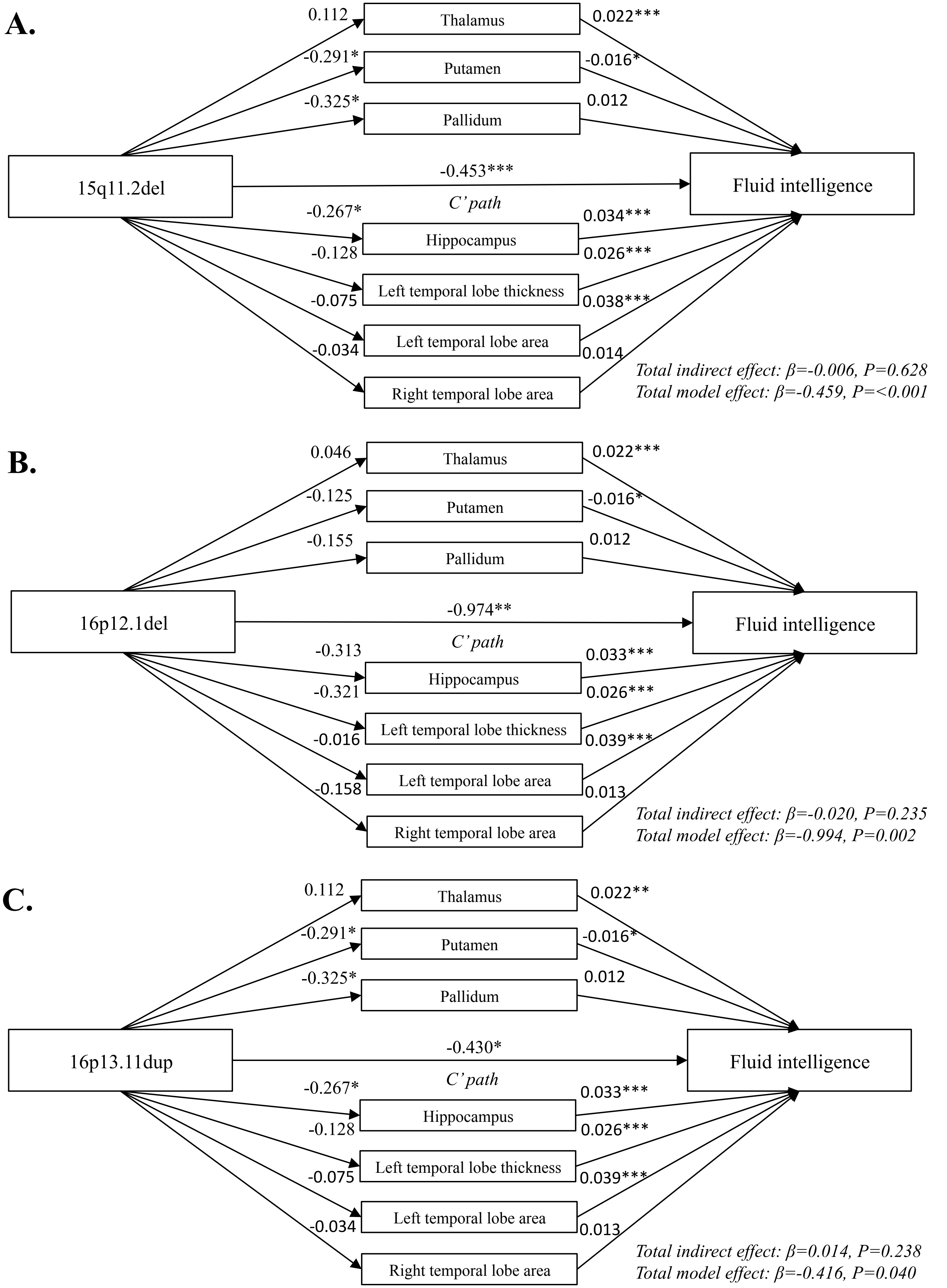


**Supplemental figure 2**. Mediation analyses for the fluid intelligence task and 15q11.2del (Figure A), 16p12.1del (Figure B), and 16p13.11dup (Figure C). Associations are presented as Beta values and significance denoted by * (p<0.05), **(p<0.01), ***(p<0.001). The C' path refers to the direct effect.

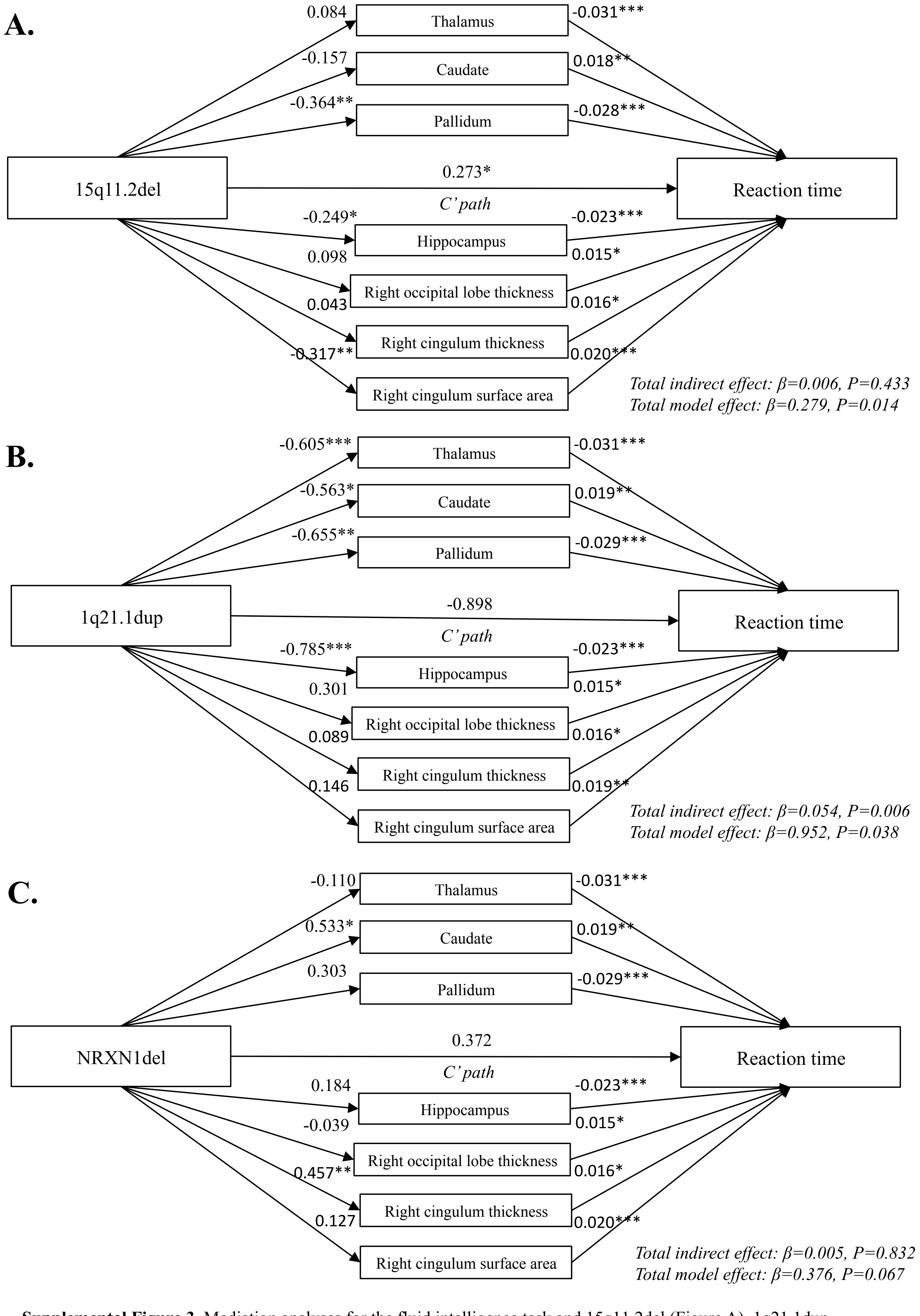


**Supplemental Figure 3**. Mediation analyses for the fluid intelligence task and 15q11.2del (Figure A), 1q21.1dup (Figure B), and NRXN1del (Figure C). Associations are presented as Beta values and significance denoted by * ($p<0.05$), **($p<0.01$), ***($p<0.001$). The C' path refers to the direct effect.
